# Quality assurance assessment of a specialized perinatal mental health clinic

**DOI:** 10.1186/s12884-020-03174-6

**Published:** 2020-08-24

**Authors:** Luisa Caropreso, Sarah Saliba, Lindsay Hasegawa, Jack Lawrence, Caitlin J. Davey, Benicio N. Frey

**Affiliations:** 1 Women’s Health Concerns Clinic, St Joseph’s Healthcare Hamilton, Hamilton, ON Canada; 2 Department of Psychiatry & Behavioural Neurosciences, McMaster University, Hamilton, ON Canada; 3 Michael G. DeGroote School of Medicine, McMaster University, Hamilton, Ontario Canada; 4 Department of Obstetrics and Gynaecology, University of Toronto, Toronto, Ontario Canada; 5 Mood Disorders Program, St Joseph’s Healthcare Hamilton, 100 West 5th St., Suite C124, Hamilton, Ontario L8N 3K7 Canada

**Keywords:** Program evaluation, Quality assurance, Mood disorders, Anxiety disorders, Women’s health, Perinatal period

## Abstract

**Background:**

Mood and anxiety issues are the main mental health complaints of women during pregnancy and the postpartum period. Services targeting such women can reduce perinatal complications related to psychiatric difficulties. This quality assurance project aimed to examine changes in mood and anxiety symptoms in pregnant and postpartum women referred to the Women’s Health Concerns Clinic (WHCC), a specialized outpatient women’s mental health program.

**Methods:**

We extracted patient characteristics and service utilization from electronic medical records of women referred between 2015 and 2016. We also extracted admission and discharge scores on the Edinburgh Postnatal Depression Scale (EPDS) and the Generalized Anxiety Disorder-7 (GAD-7) scale.

**Results:**

Most patients accessed the WHCC during pregnancy (54%), had a diagnosis of major depressive disorder (54.9%), were prescribed a change in their medication or dose (61.9%), and accessed psychotherapy for perinatal anxiety (30.1%). There was a significant decrease in EPDS scores between admission and discharge (*t*(214) = 11.57; *p* = .000; effect size *d* = .86), as well as in GAD-7 scores (*t*(51) = 3.63; *p* = .001; effect size *d* = .61). A secondary analysis showed that patients with more severe depression and anxiety symptoms demonstrated even greater effect sizes.

**Conclusions:**

Changes in EPDS and GAD-7 scores indicate that the WHCC is effective in reducing mood and anxiety symptoms associated with the perinatal period. This project highlights the importance of quality assurance methods in evaluating the effectiveness of clinical services targeting perinatal mental health, in order to inform policy and funding strategies.

## Background

The perinatal period is a time of physiological, mental, and emotional change for women. During this time, women are at an increased risk of developing new and worsening of pre-existing mental illnesses, such as depression [1], anxiety [2] and bipolar disorder [3].

Perinatal depression affects approximately 12% of women worldwide [4], whereas the prevalence of having at least one or more anxiety disorder during the perinatal period was estimated to be 20.7% [5]. In Canada, the prevalence of postpartum depression was estimated to be 7.4% [6, 7], and a recent survey on Maternal Mental Health indicated that 23% of postpartum Canadian women reported symptoms of depression and/or anxiety [8]. While the exact causes of perinatal mental health concerns are not fully understood, these mental health manifestations are typically influenced by biological, psychological, and/or social factors.

Perinatal mental health disorders have been associated with adverse pregnancy outcomes such as intrauterine growth restriction, low birth weight, and prematurity, as well as long-term effects on emotional, behavioural, and cognitive development in children. In severe cases, suicide or infanticide can result [9–13]. A recent 15-year population-based study of Ontario women found that suicide accounted for 5.1% of perinatal deaths [14]. Treatments for perinatal mental disorders typically involve psychotherapy, such as cognitive behavioural therapy (CBT) or interpersonal therapy (IPT), and/or pharmacotherapy for more severe cases [15, 16]. With treatment, approximately 70–80% of women recover from a perinatal mental health concern [17].

Perinatal mental health services throughout Canada help women access care in the peripartum period. In Ontario, these services are distributed within the Local Health Integration Networks. Available programs include the Women’s Health Concerns Clinic (WHCC) at St. Joseph’s Healthcare Hamilton, Mississauga’s Women’s Reproductive Mental Health Program at Credit Valley Hospital, Toronto’s Reproductive Life Stages Programme at Women’s College Hospital, and Sudbury’s Perinatal Mental Health Program at Health Sciences North [18]. Reproductive mental health resources in other provinces, such as British Columbia and Nova Scotia, tend to be linked with academic centers [19]. Despite having many Canadian clinics that specialize in the treatment of women’s mental health, a systematic literature search showed that there is a lack of evidence about quality assurance of perinatal mental healthcare services [20]. Furthermore, previous reviews on quality assurance in mental health care have identified challenges in completing these projects given the structure of many mental health clinics and the nature of mental healthcare [21]. For example, these clinics often involve a multidisciplinary team each contributing to evaluation and treatment, with various methods of data collection and documentation. There are also technical difficulties in tracking patients receiving multiple and different treatments, and a lack of consistency in assessment tools [21]. In addition, results from countries with a mix of private/publicly-insured health systems might not be applicable to the Canadian population, where health services are largely publicly insured [22].

Given the high prevalence of mental health concerns during the perinatal period as well as the previously identified challenges in evaluating the quality of women’s mental health resources, we sought to evaluate the effectiveness of the WHCC in managing perinatal mood and anxiety disorders using a standardized process for collecting patient data and to elucidate the characteristics and service access of these patients. In doing so, we aimed to examine the challenges in assuring and evaluating service quality as discussed above, while also filling a gap in the literature on the quality assurance of a perinatal mental health services.

## Methods

### Clinical setting

The WHCC is a multidisciplinary mental health clinic affiliated with the Department of Psychiatry and Behavioural Neurosciences at McMaster University, and St. Joseph’s Healthcare in Hamilton, Ontario, Canada. Women are usually referred by community providers (typically family doctors, midwives, obstetricians or community psychiatrists), and the clinic also accepts self-referrals. During the perinatal period, women are followed at the WHCC while facing any mental health issue, and they can be followed proactively even in the absence of symptoms if they are considered high risk (e.g. present with a past history of perinatal or other mental health disorders).

At the WHCC, patients are assessed by a psychiatrist and a registered nurse, social worker, or a master’s level therapist. The first consult consists of a comprehensive psychiatric assessment, and patients are typically followed throughout pregnancy and up to 9 months postpartum, according to their needs, and then referred back to their family doctor/community psychiatrist.

Regarding treatment offered at the WHCC, the clinic offers four non-pharmacological treatment options in group format during the perinatal period: CBT for perinatal anxiety [23], CBT for perinatal depression [24], psychoeducation for perinatal bipolar disorder, and emotional regulation skills. If necessary, patients may also access individual therapy, which may include CBT or supportive counselling. Although psychotherapy does not have a uniform public funding coverage across Canada, all the services offered at the WHCC are publicly covered by the Ontario Health Insurance Program. Pharmacological treatment through consultation and close follow-up with a psychiatrist is also available. Discussions regarding risks and benefits of pharmacological treatment typically include potential risks of no treatment versus obstetric and neonatal risks [25, 26]. It is standard practice at the WHCC to provide all patients with such information. At the WHCC, patients are also provided with information on community and online resources for further education. In addition, the standard of care at WHCC involves measurement-based care with the use of standardized clinical questionnaires validated on their own clinical population [27, 28].

### Data extraction

The following data were extracted by 5 team members (CD, JL, LH, LC, SS) from the electronic medical records of 226 patients who completed the clinical questionnaires at admission and discharge between January 2015–December 2016: Age, marital status, pregnancy status at service entry (i.e., pregnant or postpartum), psychiatric diagnosis, services used at the WHCC, use of medications (including changes made throughout their course of treatment) and the Edinburgh Postnatal Depression Scale (EPDS) [29], and/or the Generalized Anxiety Disorder Scale (GAD-7) [30] scores at first assessment and discharge. In order to be included in this service evaluation analysis, patients had to be either pregnant or postpartum, have had at least two visits at the clinic, and have had at least two GAD-7/EPDS measurements. Participants were excluded from the analysis if they were seen for pregnancy planning consultations only, if they were lost to follow-up after the first visit, or if they had only one GAD-7/EPDS measurement available.

### Ethics

The local Hamilton Integrated Research Ethics Board was consulted to determine the need for ethics approval. Due to the fact that this project was designed as a quality assurance service evaluation, we received written communication from the Ethics Board that an ethics submission was not deemed necessary. Our team received administrative permission to access the data used in this research for quality assurance purposes.

### Measurements

At the first consult at the WHCC, pregnant/postpartum patients are asked to complete the Mood Disorder Questionnaire (MDQ [27]), the Perinatal Obsessive-Compulsive Scale (POCS) [31], the EPDS and the GAD-7. In addition, the EPDS and GAD-7 are repeated in each follow-up visit. These instruments have good psychometric properties, have been locally validated and have well-established clinical cut-offs for our specific population seen at the WHCC [28]. Both of these questionnaires are not only well-established screening tools, but they are also sensitive to track treatment response.

The EPDS is a self-report questionnaire containing 10 questions regarding how the patient has felt in the past 2 weeks, and can be rated from 0 to 30. The sensitivity of the EPDS at a cut-off score of ≥13 was estimated to be approximately 86% and specificity was about 78%, with a positive predictive value of 73% [29, 32, 33].

The GAD-7 has 7 questions, with total scores ranging from 0 to 21 [30]. The GAD-7 has been tested in a local population of 240 perinatal women (*n* = 155 pregnant and *n* = 85 postpartum), yielding a sensitivity of 0.61 and specificity of 0.72 at an optimal cut-off score of 13 [28].

### Data analysis

Clinical data were analyzed using Statistical Package for the Social Sciences (SPSS) Version 25 statistical software. Patient characteristics (e.g., patient age, perinatal status), psychiatric diagnoses, and treatment type (i.e., pharmacotherapy and psychotherapy) were analyzed with descriptive statistics. Pre-treatment and post-treatment EPDS and GAD-7 data were examined for violation of assumptions, including normal distributions of the score differences. Assumptions were not violated. Pre-post comparisons for both the EPDS and GAD-7 were completed using dependent t-tests. The *p*-value used for significance was .05 and effect sizes were calculated using Cohen’s *d*. An effect size of .20 is considered small, of .50 is considered medium and .80 is considered large [34]. In order to test whether length of treatment had an impact on changes in EPDS or GAD-7 scores, length of treatment was entered as a covariate using repeated measures analysis of variance (ANOVA). In the current study we used the same EPDS and GAD-7 cut-offs that have been recommended in the literature (EPDS/GAD-7 score of ≥13).

## Results

### Patient demographics and mental health diagnoses

Patients accessing the WHCC ranged in age from 18 to 42 years, with a mean age of 30.9 years. The majority of patients seen at the WHCC were pregnant at baseline (54%) and the rest were postpartum (44.2%). The majority of patients were partnered/married (83.2%; Table 1). Most patients accessing the WHCC had a diagnosis of major depressive disorder (54.9%). Other diagnoses included generalized anxiety disorder (43.8%), posttraumatic stress disorder (8%), social anxiety disorder (10.2%), other anxiety disorders (e.g., anxiety Not Otherwise Specified, panic attacks, and specific phobia; 13.7%), bipolar disorder (I or II; 8%), obsessive-compulsive disorder (10.2%), borderline personality (including traits; 7.1%), substance use disorder (5.8%), eating disorders (4%), other personality disorders (includes traits; 2.2%), and other (e.g., trichotillomania, psychosis, attention-deficit and hyperactivity disorder, adjustment disorder, learning disability; 9.3%). Most patients had at least one comorbid diagnosis (i.e., 27.9% were diagnosed with major depressive disorder and generalized anxiety disorder, while 31.9% had other comorbidities; Table 2).

**Table 1:** Patient Demographics (*n* = 226)

	Mean ± SD
***Age (years)***	30.9 ± 5.1
***Perinatal Status***	**N (%)**
Pregnant	122 (54%)
Postpartum	100 (44.2%)
Missing	4 (1.8%)
***Relationship Status***	**N (%)**
Single/Divorced/Separated	30 (13.3%)
Partnered/Married	188 (83.2%)
Missing	8 (3.5%)

**Table 2:** Mental Health Diagnoses (*n* = 226)

	N (%)		N (%)
***Diagnoses***		***Comorbidities***	
Unipolar Depression (Major Depressive, Persistent Depressive Disorders)	124 (54.9%)	Major Depressive Disorder + Generalized Anxiety Disorder	63 (27.9%)
Generalized Anxiety Disorder	99 (43.8%)	Other Comorbidities	72 (31.9%)
Other Anxiety Disorders (Anxiety Not Otherwise Specified, panic attacks, Specific Phobia	31 (13.7%)	No Comorbidities	79 (35%)
Bipolar Disorder (Type I or II)	18 (8%)	Missing	12 (5.3%)
Post-Traumatic Stress Disorder	18 (8%)		
Obsessive Compulsive Disorder	23 (10.2%)		
Borderline Personality (including traits)	16 (7.1%)		
Social Anxiety Disorder	23 (10.2%)		
Substance Use Disorder	13 (5.8%)		
Eating Disorder	9 (4%)		
Other Personality Disorders (including traits)	5 (2.2%)		
Other (trichotillomania, psychosis, attention-deficit and hyperactivity disorder, adjustment disorder, learning disability	21 (9.3%)		

### Patient treatment and initial service access

Upon admission, most patients were prescribed a new psychotropic medication or increased the dosage of medication they had already been taking (61.9%), and most patients were referred to psychotherapy services, including CBT for perinatal anxiety (30.1%), individual therapy (including supportive counselling and individual CBT; 20.8%). Patients also attended CBT for depression (8.4%) and psychoeducation for bipolar disorder (2.7%). It should be noted that 6.6% of WHCC patients were referred for psychotherapy services through the Mood Disorders Clinic at St. Joseph’s Healthcare Hamilton, which is a clinic that works very closely with the WHCC, and 2.7% accessed an external service (Table 3). The percentages of those who accessed the treatments listed above during pregnancy were similar to those who accessed such treatments during the postpartum period. The average length of time that patients received treatment at the WHCC (i.e., from first assessment to discharge) was 227 days (SD = 171.76 days; Mode = 196 days) and the average number of CBT sessions that patients attended (individual and/or group-based) were 4.6 sessions (SD = 3; Mode = 6 sessions).

**Table 3:** WHCC Service Access (*n* = 226)

	N (%)		N (%)
***Psychotherapy***		***Pharmacotherapy***	
CBT for Perinatal Anxiety	68 (30.1%)	No change in medications	70 (31%)
CBT for Perinatal Depression	19 (8.4%)	New medication/increased dose	140 (61.9%)
Psychoeducation for Bipolar Disorder	6 (2.7%)	Stopped medication/decreased dose	7 (3.1%)
Individual Therapy (Including CBT or Supportive Therapy)	47 (20.8%)	Missing	9 (4%)
Mood Disorders Clinic Services	15 (6.6%)		
External Services	6 (2.7%)		
Missing	65 (28.8%)		

### Changes in EPDS and GAD-7 scores

A total of *n* = 215 pre and post EPDS and *n* = 52 pre and post GAD-7 data were extracted. There was a significant decrease in EPDS scores: *t*(214) = 11.57, *p* = .000 with a large effect size of *d* = .86, as well as a significant decrease in GAD-7 scores: *t*(51) = 3.63, *p* = .001 with a medium effect size of *d* = .61.

We conducted a secondary analysis including only those who scored above the clinical cut-off of 13 for the EPDS and GAD-7 at baseline (i.e., those with more severe depressive and anxiety symptoms). There was a significant decrease in EPDS scores: *t*(134) = 13.88, *p* = .000 with a large effect size of *d* = 1.54), and a significant decrease in GAD-7 scores (*t*(26) = 5.17, *p* = .000 with a large effect size of *d* = 1.44) among those with severe symptoms (Table 4). Repeated measures ANOVA with length of treatment as a covariate showed that length of treatment did not have any impact on changes in EPDS or GAD-7 scores.

**Table 4:** EPDS (*n* = 215) and GAD-7 (*n* = 52) scores from pre- to post- WHCC access

	Pre-Treatment Mean ± SD	Post-Treatment Mean ± SD	Statistic	p	Cohen’s ***d***
**EPDS**	14.50 ± 6.10	9.46 ± 5.67	t = 11.57	< 0.001	0.86
**GAD-7**	11.77 ± 6.19	8.14 ± 5.71	t = 3.63	0.001	0.61
**EPDS (including only those above the clinical cutoff ≥ 13;** ***n*** **= 135)**	18.34 ± 3.49	11.01 ± 5.73	t = 13.88	< 0.001	1.54
**GAD-7 (including only those above the clinical cutoff ≥ 13;** ***n*** **= 27)**	16.44 ± 2.49	9.78 ± 6.05	t = 5.17	< 0.001	1.44

## Discussion

In this quality assurance project, we reviewed the electronic medical records of perinatal patients seen at the WHCC from 2015 to 2016 and aimed to describe patient characteristics, including psychiatric diagnoses and treatment access as well as the effectiveness of a specialized perinatal mental health program in reducing symptoms of perinatal depression and anxiety. As perinatal anxiety and depression are the most common reasons for referral, we evaluated baseline and discharge GAD-7 and EPDS scores as the main outcomes.

Most patients referred to the WHCC had a diagnosis of major depressive disorder (54.9%) or generalized anxiety disorder (43.8%), with most exhibiting at least one comorbid mental health diagnosis (e.g., 27.9% of patients presented with comorbid major depressive disorder and generalized anxiety disorder). This finding is consistent with another Canadian study conducted in a perinatal psychiatric clinic, which reported that most women also had a diagnosis of either depression or anxiety, with a comorbidity rate between major depressive episode and generalized anxiety disorder of 29.7% [35]. As expected, given that the WHCC is a tertiary specialized treatment program, the rates of mood and anxiety disorders in our study were higher than those found in studies drawn from community samples [36, 37].

Most patients were prescribed a new psychotropic medication or had the dosage of a medication they had already been taking increased. This finding is comparable to a study that evaluated the rates of women using mental health services in Switzerland during the perinatal period, which showed that medication was the most frequent treatment used, with 42% of women being medicated during pregnancy and 55% of women being medicated during the postpartum period [38]. The latest Canadian guidelines recommend psychotherapy (e.g. CBT, IPT) as first-line treatment for mild to moderate depression, while medications are first-line for patients with more severe symptoms [16]. Thus, our results, showing that most women were prescribed both medications and psychotherapy are also in line with current treatment guidelines. There is a large amount of evidence showing that CBT is effective in the perinatal population (e.g., [39–41]), including data from WHCC [24, 42].

The main results of our study were the significant decrease in EPDS and GAD-7 scores from pre to post-treatment with medium to large effect sizes. The effect sizes were even larger in women presenting with more severe depressive and anxiety symptoms at intake. We attribute these successful treatment response rates to the fact that WHCC is an academic, specialized perinatal mental health service that follows evidence-based treatment guidelines, including measurement-based care, as well as the fact that WHCC has a multidisciplinary team of staff including psychiatrists, psychologists, nurses, social workers and psychotherapists [43]. Considering the high prevalence of mental health issues faced by women during the perinatal period, this quality assurance project may help to inform policy makers to improve access and treatment outcomes in this vulnerable population.

The results of this quality assurance study should be interpreted in view of its limitations. One of the limitations is that we only included patients with at least two visits, and the exclusion from the analyses of patients who did not return for a follow up visit after the initial assessment did not capture the entire clinical presentation of patients who are referred to the WHCC. Unfortunately, certain socio-demographic characteristics such as ethnicity and educational background were not regularly cited in the patients’ medical charts and could not be extracted. The relatively small number of individuals with pre and post GAD-7 scores is another limitation. The inclusion of a comparison group who did not access the support from WHCC would have strengthened the results. In addition, given the variety of combination of different treatments, a more detailed picture of treatment experiences was not possible. These limitations illustrate some of the challenges in assuring and evaluating service quality. In line with previous reports [20, 21], we also experienced difficulties in tracking the details of different combination of treatments received by the patients, as well as the lack of consistency in the documentation of certain socio-demographic characteristics. Given the paucity of publications on quality assurance of perinatal mental health services, we encourage other services to perform quality assurance projects and disseminate their findings outside the institution walls.

## Conclusions

One in 10 women suffers from clinically significant depressive and/or anxiety symptoms during the perinatal period. We showed that a specialized perinatal outpatient multidisciplinary team program that follows evidence-based guidelines and measurement-based care can significantly decrease mental health suffering in this population. Results from this study can inform healthcare stakeholders, policy makers and funding strategies for the publicly-funded health care systems.

## Data Availability

We cannot share the raw data used in this study because this is a quality assurance project where we did not obtain consent from participants for data sharing.

## Abbreviations

ANOVA: Analysis of variance
CBT: Cognitive behavioural therapy
EPDS: Edinburgh Postnatal Depression Scale
GAD-7: Generalized Anxiety Disorder-7
IPT: Interpersonal therapy
MDQ: Mood Disorder Questionnaire
POCS: Perinatal Obsessive-Compulsive Scale
SPSS: Statistical Package for the Social Sciences
WHCC: Women’s Health Concerns Clinic

## References

[1] Bennett HA, Einarson A, Taddio A, Koren G, Einarson TR (2004). Prevalence of depression during pregnancy: systematic review. Obstet Gynecol.

[2] Ross LE, McLean LM (2006). Anxiety disorders during pregnancy and the postpartum period: a systematic review. J Clin Psychol.

[3] Viguera AC, Whitfield T, Baldessarini RJ, Newport DJ, Stowe Z, Reminick A (2007). Risk of recurrence in women with bipolar disorder during pregnancy: prospective study of mood stabilizer discontinuation. Am J Psychiatry.

[4] Woody CA, Ferrari AJ, Siskind DJ, Whiteford HA, Harris MG (2017). A systematic review and meta-regression of the prevalence and incidence of perinatal depression. J Affect Disord.

[5] Fawcett EJ, Fairbrother N, Cox ML, White IR, Fawcett JM (2019). The prevalence of anxiety disorders during pregnancy and the postpartum period: a multivariate Bayesian meta-analysis. J Clin Psychiatry.

[6] Daoud N, O’Brien K, O’Campo P, Harney S, Harney E, Bebee K, Smylie J (2019). Postpartum depression prevalence and risk factors among indigenous, non-indigenous and immigrant women in Canada. Can J Public Health.

[7] Vigod SN, Tarasoff LA, Bryja B, Dennis CL, Yudin MH, Ross LE (2013). Relation between place of residence and postpartum depression. Can Med Assoc J.

[8] Statistic Canada, Canada: https://www150.statcan.gc.ca/n1/pub/11-627-m/11-627-m2019041-eng.htm [November 1^st^, 2019].

[9] Stein A, Pearson R, Goodman S, Rapa E, Rahman A, McCallum M (2014). Effects of perinatal mental disorders on the fetus and child. Lancet.

[10] Dennis CL (2004). Influence of depressive symptomatology on maternal health service utilization and general health. Arch Womens Ment Health.

[11] Field T, Diego M, Hernandez-Reif M, Schanberg S, Kuhn C, Yando R (2003). Pregnancy anxiety and comorbid depression and anger: effects on the fetus and neonate. Depress Anxiety.

[12] Henrichs J, Schenk JJ, Roza SJ, van den Berg MP, Schmidt HG, Steegers EA (2010). Maternal psychological distress and fetal growth trajectories: the generation R study. Psychol Med.

[13] van den Berg MP, van der Ende J, Crijnen AA, Jaddoe VW, Moll HA, Mackenbach JP (2009). Paternal depressive symptoms during pregnancy are related to excessive infant crying. Pediatrics.

[14] Grigoriadis S, Wilton AS, Kurdyak PA, Rhodes AE, VonderPorten EH, Levitt A (2017). Perinatal suicide in Ontario, Canada: a 15-year population-based study. Can Med Assoc J.

[15] Howard L, Molyneaux E, Dennis C, Rochat T, Stein A, Milgrom J (2014). Non-psychotic mental disorders in the perinatal period. Lancet.

[16] MacQueen GM, Frey BN, Ismail Z, Jaworska N, Steiner M, Van Lieshout RJ (2016). Canadian network for mood and anxiety treatments (CANMAT) 2016 clinical guidelines for the management of adults with major depressive disorder: section 6. Special populations: youth, women, and the elderly. Can J Psychiatr.

[17] Ross LE, Dennis CL, Blackmore ER, Stewart DE (2005). Postpartum depression: a guide for front-line health and social service providers.

[18] Echo: Improving Women’s Health in Ontario (2012). The organization of perinatal mental health services in Ontario: recommendations for service, education and training, policy and research.

[19] Best practice guideline for mental health disorder in perinatal period: http://www.perinatalservicesbc.ca/Documents/Guidelines-standards/Maternal/MentalHealthDisordersGuideline.pdf [May 3rd, 2019].

[20] Gaebel W, Großimlinghaus I, Heun R, Janssen B, Johnson B, Kurimay T (2015). European psychiatric association (EPA) guidance on quality assurance in mental healthcare. Eur Psychiat.

[21] Zayas L, McMillen J, Lee M, Books S (2011). Challenges to quality assurance and improvement efforts in behavioral health organizations: a qualitative assessment. Admin Pol Ment Health.

[22] Health Canada, Canada: https://www.canada.ca/en/health-canada/services/health-care-system/reports-publications/health-care-system/canada.html [Novermber 1st, 2019].

[23] Green SM, Frey BN, Donegan E, McCabe RE (2019). Cognitive behavioral therapy for anxiety and depression during pregnancy and beyond: how to manage symptoms and maximize well-being.

[24] Van Lieshout RJ, Yang L, Haber E, Ferro MA (2016). Evaluating the effectiveness of a brief group cognitive behavioural therapy intervention for perinatal depression. Arch Women Ment Health.

[25] Antenatal and postnatal mental health: clinical management and service guidance, National Institute for Health and Care Excellence: https://www.nice.org.uk/guidance/CG192 [May 3rd, 2019]. 31990493

[26] McAllister-Williams RH, Baldwin DS, Cantwell R, Easter A, Gilvarry E, Glover V (2017). British Association for Psychopharmacology consensus guidance on the use of psychotropic medication preconception, in pregnancy and postpartum. J Psychopharmacol.

[27] Frey BN, Simpson W, Wright L, Steiner M (2012). Sensitivity and specificity of the mood disorder questionnaire as a screening tool for bipolar disorder during pregnancy and the postpartum period. J Clin Psychiatry.

[28] Simpson W, Glazer M, Michalski N, Steiner M, Frey BN (2014). Comparative efficacy of the generalized anxiety disorder 7-item scale and the Edinburgh postnatal depression scale as screening tools for generalized anxiety disorder in pregnancy and the postpartum period. Can J Psychiatr.

[29] Cox JL, Holden JM, Sagovsky R (1987). Detection of postnatal depression. Development of the 10-item Edinburgh postnatal depression scale. Br J Psychiatry.

[30] Spitzer RL, Kroenke K, Williams JB, Löwe B (2006). A brief measure for assessing generalized anxiety disorder: the GAD-7. Arch Intern Med.

[31] Lord C, Rieder A, Hall GBC, Soares CN, Steiner M (2011). Piloting the perinatal obsessive-compulsive scale (POCS): development and validation. J Anxiety Disord.

[32] O’Connor E, Rossom RC, Henninger M, Groom HC, Burda BU (2016). Primary care screening for and treatment of depression in pregnant and postpartum women. J Am Med Assoc.

[33] Flynn HA, Sexton M, Ratliff S, Porter K, Zivin K (2011). Comparative performance of the Edinburgh postnatal depression scale and the patient health Questionnaire-9 in pregnant and postpartum women seeking psychiatric services. Psychiatry Res.

[34] Cohen J (1988). Statistical power analysis for the behavioral sciences.

[35] Grigoriadis S, de Camps MD, Barrons E, Bradley L, Eady A, Fishell A (2011). Mood and anxiety disorders in a sample of Canadian perinatal women referred for psychiatric care. Arch Women Ment Health.

[36] Vesga-López O, Blanco C, Keyes K, Olfson M, Grant BF, Hasin DS (2008). Psychiatric disorders in pregnant and postpartum women in the United States. Arch Gen Psychiatry.

[37] Wenzel A, Haugen EN, Jackson LC (2005). Anxiety symptoms and disorders at eight weeks postpartum. J Anxiety Disord.

[38] Berger A, Bachmann N, Signorell A, Erdin R, Oelhafen S, Reich O (2017). Perinatal mental disorders in Switzerland: prevalence estimates and use of mental-health services. Swiss Med Wkly.

[39] Le H, Perry DF, Stuart EA (2011). Randomized controlled trial of a preventive intervention for perinatal depression in high-risk Latinas. J Consult Clin Psychol.

[40] Loughnan SA, Wallace M, Joubert AE, Haskelberg H, Andrews G, Newby JM (2018). A systematic review of psychological treatments for clinical anxiety during the perinatal period. Arch Women Ment Hlth.

[41] O’Mahen H, Himle JA, Fedock G, Henshaw E, Flynn H (2013). A pilot randomized controlled trial of cognitive behavioral therapy for perinatal depression adapted for women with low incomes. Depress Anxiety.

[42] Green SM, Donegan E, McCabe RE, Streiner DL, Agako A, Frey BN (2020). Cognitive behavioral therapy for perinatal anxiety: a randomized controlled trial. Aust N Z J Psychiatry.

[43] Women’s Health Concerns Clinic, Canada: www.stjoes.ca/whcc [November 1^st^, 2019].

